# Somatostatin receptors 2 and 5 are preferentially expressed in proliferating endothelium

**DOI:** 10.1038/sj.bjc.6602503

**Published:** 2005-04-05

**Authors:** R L Adams, I P Adams, S W Lindow, W Zhong, S L Atkin

**Affiliations:** 1 Endocrinology Research Group, Division of Academic Medicine, University of Hull Postgraduate Medical School, Hull HU6 7RX, UK; 2 Department of Obstetrics and Gynecology, Hull Women and Children's Hospital, Hull HU3 2JZ, UK

**Keywords:** somatostatin receptors, endothelium, angiogenesis, proliferation, Octreotide, SOM230

## Abstract

Angiogenesis is characterised by activation, migration and proliferation of endothelial cells and is central to the pathology of cancer, cardiovascular disease and chronic inflammation. Somatostatin is an inhibitory polypeptide that acts through five receptors (sst 1, 2, 3, 4, 5). Sst has previously been reported in endothelium, but their role remains obscure. Here, we report the expression of sst in human umbilical vein endothelial cells (HUVECs) *in vitro*, during proliferation and quiescence. A protocol for culturing proliferating and quiescent HUVECs was established, and verified by analysing cell cycle distribution in propidium-iodide-stained samples using flow cytometry. Sst mRNA was then quantified in nine proliferating and quiescent HUVEC lines using quantitative reverse transcriptase–polymerase chain reaction. Sst 2 and 5 were preferentially expressed in proliferating HUVECs. All samples were negative for sst 4. Sst 1 and 3 expression and cell cycle progression were unrelated. Immunostaining for sst 2 and 5 showed positivity in proliferating but not quiescent cells, confirming sst 2 and 5 protein expression. Inhibition of proliferating cells with somatostatin analogues Octreotide and SOM230, which have sst 5 activity, was found (Octreotide 10^−10^–10^−6^ M: 48.5–70.2% inhibition; SOM230 10^−9^–10^−6^ M: 44.9–65.4% inhibition) in a dose-dependent manner, suggesting that sst 5 may have functional activity in proliferation. Dynamic changes in sst 2 and 5 expression during the cell cycle and the inhibition of proliferation with specific analogues suggest that these receptors may have a role in angiogenesis.

Angiogenesis is a carefully regulated normal physiologic process, required for wound healing, reproduction and development (Griffioen and Molema, 2000). Vascular endothelial cells are normally quiescent cells, dividing every 2–5 years (Woltering *et al*, 1997). However, endothelial cell stimulation by proangiogenic factors and cytokines from inflammatory cells or tumours results in their proliferation with an altered expression profile (Fox *et al*, 2001).

Somatostatin is a widely distributed inhibitory polypeptide that inhibits exocrine secretion, cellular proliferation and cellular differentiation, and promotes apoptosis. The effects of Somatostatin and its analogues are mediated via five G-protein-linked receptors (sst 1, 2, 3, 4, 5). These receptors act through multiple signal transduction pathways to elicit their inhibitory effects.

Proliferating endothelium *in vitro* express sst 2 that is also expressed by endothelial cells within or adjacent to tumours (Reubi *et al*, 1994, 1996, 2001; ten Bokum *et al*, 1999, Watson *et al*, 2001; Koizumi *et al*, 2002), and it has been shown that sst 2 is expressed in the angiogenic sprouts of endothelium from placental veins (Watson *et al*, 2001). Experimental angiogenesis has been inhibited by the synthetic sst analogue Octreotide (Woltering *et al*, 1991; Danesi and Del Tacca, 1996; Danesi *et al*, 1997) that has a high affinity for sst 2. Human umbilical vein endothelial cells (HUVECs), which are widely used as an endothelial cell model *in vitro*, have also been shown to express sst 2 (Curtis *et al*, 2000).

Sst 1 has previously been detected in HUVECs (Curtis *et al*, 2000), and in the endothelium of neuroblastomas (Albers *et al*, 2000) and benign and malignant ovarian tumours (Hall *et al*, 2002). Sst 3 expression has also been demonstrated in HUVECs (Jia *et al*, 2003), and in the endothelial cell line Eahy926 (Florio *et al*, 2003). Sst 4 has only been reported in HUVECs (Curtis *et al*, 2000). There are currently no reports in the literature indicating that sst 1, 3 or 4 expression is unique to proliferating vessels. The expression of sst 5 in primary human endothelial cells remains unreported.

Therefore, with the knowledge of the selective expression of sst 2 in proliferating endothelial cells, the aim of these studies was to determine whether there was a change in sst 1, 3, 4 and 5 expression in proliferating *vs* quiescent human umbilical endothelial cells *in vitro*.

## MATERIALS AND METHODS

### HUVEC isolation and culture

Human umbilical cords were obtained from Hull Maternity Hospital with permission from the local ethics committee and informed patient consent. HUVECs were harvested by collagenase digestion, according to the method by Jaffe *et al* (1973). Reagents were purchased from Invitrogen (Paisley, UK), unless otherwise stated. Cells were routinely cultured in D-MEM with 5 mM D-glucose supplemented with 20% foetal bovine serum (FBS), 20 ng ml^−1^ human *β*-endothelial cell growth factor (Sigma, Poole, UK), 8 U ml^−1^ heparin sodium salt (Sigma), 2 mM L-glutamine, 50 U ml^−1^ penicillin G and 50 *μ*g ml^−1^ streptomycin sulphate. Culture vessels were coated in 1% gelatin (Sigma) for 10 min prior to use. The HUVECs were grown at 37°C with 5% CO_2_ in a humidified environment. Immunohistochemical detection of von Willebrand factor confirmed that the cells were of endothelial origin. Cells were passaged at a ratio of 1 : 3 after reaching 80–90% confluence, and media were replenished every 48 h.

### Establishment of proliferating and quiescent HUVECs

In all, 14 separate HUVECs lines derived from different patients were seeded into six-well plates at a density of 10^5^ cells well^−1^ in 2 ml of medium. Cells that approximately doubled in number within 24 h, as determined by microscopic examination, were tentatively identified as proliferating cells. Quiescent cells were left to grow until confluent and then the medium was replaced with growth factor-free medium for 24 h. Growth factor-free medium lacked ECGF and heparin sodium salt, and FBS was replaced with charcoal-stripped FBS. The viability of quiescent cells was demonstrated by passaging them at a 1 : 2 ratio after 48 h growth factor deprivation, and then confirming that they grew to confluence again within 48 h. The proliferative status of the cells was confirmed using cell cycle analysis as described below. Nine of the lines of proliferating and quiescent HUVECs were grown in triplicate for RNA extraction. The three lines were used for cell cycle analysis alone, one line was used for the immunohistochemistry and another to determine the effects of the somatostatin analogues Octreotide and SOM230 on HUVEC proliferation.

### HUVEC cell cycle analysis

Propidium-iodide-stained samples were analysed for cell cycle distribution as described previously (Newton *et al*, 2003). Briefly, HUVECs from six-well plates were centrifuged at 200 **g** for 10 min, resuspended in 300 *μ*l phosphate-buffered saline (PBS) and then fixed in 70% ice-cold ethanol overnight. The cells were recovered by centrifugation, and resuspended in 250 *μ*l of PBS to which 10 *μ*l of 0.5 mg ml^−1^ propidium iodide solution was added. Following the addition of propidium iodide, the samples underwent incubation for 30 min at 37°C. Samples were analysed with a FACS^CALIBUR^ flow cytometer (Becton Dickinson, Cowley, UK) with an argon laser tuned to 488 nm. Forward and orthogonal light scatter and red fluorescence (FL-2) were then determined from at least 10 000 events. Histogram plots were analysed using the cell cycle analysis software, Modfit (Becton Dickinson).

### Quantitative reverse transcriptase–real-time polymerase chain reaction (qRT–PCR)

Primer sets were then submitted to a BLAST search at http://www.ncbi.nlm.nih.gov/BL AST/ to confirm their uniqueness. The specificity of each primer set was confirmed in the results for the relative expression of sst in proliferating and quiescent HUVECs, where the detection of any one sst does not depend on the detection of another. RNA was extracted from HUVECs grown in six-well plates using Trizol (Invitrogen), as directed. Since sst are intronless, RNA from each sample was treated with deoxyribonuclease I (Invitrogen) to remove contaminating genomic DNA prior to reverse transcription, in the presence of RNAsin ribonuclease inhibitor (Promega UK Ltd, Southampton, UK). The RNA was reverse transcribed with Moloney murine leukaemia virus reverse transcriptase using random primers (Invitrogen), according to the manufacturer's instructions. qRT–PCR was performed utilising the ABI prism 5700 sequence detection system (Applied Biosystems, Warrington, UK). Sst 1–5 oligonucleotide forward primers, reverse primers and internal probes were designed using Primer Express version 1.0 (Applied Biosystems), and synthesised by MWG-Biotech (Ebersberg, Germany). The internal probes were labelled at the 5′-ends with the reporter fluorochrome 6-carboxyfluorescein and at the 3′-ends with the quencher fluorochrome 6-carboxytetramethylrhodamine. These primers and probes are detailed in Table 1. Each reaction volume was 25 *μ*l, and contained 1 × TaqMan Universal Master Mix (Applied Biosystems), 5 *μ*l cDNA, 300 nM forward primer, 300 nM reverse primer and 150 nM internal probe. Amplification of the human *β*-glucoronidase housekeeping gene (Applied Biosystems) was used as an internal standard. Water was used as a nontemplate control. Nonreverse-transcribed samples were run in parallel to confirm that positive results were not due to amplification of genomic DNA. Human genomic DNA was used as a positive control for all sst. The PCR cycle consisted of an initial cycle of 50°C for 2 min followed by 95°C for 10 min, and then 50 repeated cycles of 95°C for 15 s (denaturation) and 60°C for 1 min (primer annealing and extension). Proliferating and quiescent HUVECs that were to be compared were assayed simultaneously to ensure accurate relative quantification as described previously (Green *et al*, 2002).

### Immunohistochemistry

Proliferating and quiescent cells were cultured as above in Labtec chamber slides in quadruplicate (Nunc, Wiesbaden, Germany). The media were removed and the cells were fixed in 95% ice-cold ethanol for 60 min, followed by washing in PBS three times. Immunocytochemistry was performed for sst 2 and 5 as detailed previously (Stafford *et al*, 2004). Briefly, nonspecific binding sites were blocked with an Avidin/Biotin Blocking Kit (Vector Ltd, UK) and nonspecific serum protein block and endogenous peroxidase activity was quenched by incubating the cells with 1% H_2_O_2_. Rabbit monoclonal antibodies to sst 2 and 5 were obtained from Gramsch Laboratories (Schwabhausen, Germany). Sections were then incubated overnight at 4°C with primary antibody, diluted to 1 : 10 000 in PBS plus 1% bovine serum albumin and 0.3% Triton X-100.

Signal from the bound primary antibody was then amplified and visualised using the DAKO Catalysed Signal Amplification Peroxidase System K1500 (Dako, High Wycombe, Bucks, UK). The streptavidin/biotin complex was applied and signal amplified by adding the amplification reagent prior to streptavidin peroxidase. Immunoreactivity was then visualised by adding hydrogen peroxide as the enzyme substrate, in the presence of 0.05% 3,3′-diaminobenzidine. Signal was intensified with copper sulphate and nuclei lightly counterstained with Harris haematoxylin, before rehydrating and mounting with DPX.

Samples of anterior pituitary and normal pancreas known to express the relevant antigen were used as positive controls. Negative controls included omission of the primary antibody and incubation with 1% nonimmune serum.

### Effect of the somatostatin analogues Octreotide and SOM230

To determine whether sst may be functionally important in proliferation, dose–response curves were constructed with the somatostatin analogues Octreotide and SOM230 (a gift from Novartis Ltd, Basle, Switzerland). Octreotide is a somatostatin analogue in clinical practice that has affinity for sst 2 and 5 (0.38±0.08 and 6.3±1.0 IC_50_ (nM)±s.e.m., respectively), while SOM230 is an experimental multiligand receptor analogue that has affinity for sst 1, 2, 3 and 5 (9.3±0.1, 1.0±0.1, 1.5±03 and 0.16±0.01 IC_50_ (nM)±s.e.m., respectively). HUVEC proliferation was assessed using the WST-1 proliferation assay (Roche, Lewes, UK). HUVECs were aliquoted into gelatin-coated 96-well culture plates at a density of 2500 cells−well^−1^. After 18 h incubation, the medium was replaced with fresh medium containing 20% charcoal-stripped FBS, and excluding regular FBS and ECGF. At 6 h after the medium was switched, HUVECs were treated with 10^−6^–10^−10^ M SOM230 and Octreotide in parallel for 21 h. For the final 3 h of incubation, WST-1 was added to each well according to the manufacturer's instructions, and absorbance measured hourly for 3 h, at a wavelength of 450 nm with a reference wavelength of 620 nm using an Anthos 2010 plate reader (Anthos-Labtec, Salzburg, Austria). To ensure that sst 5 was expressed under these conditions, immunostaining for sst 5 was undertaken and shown to be positive. Six replicates were performed for each point of the dose–response curves.

### Statistical analysis

The data were analysed with SPSS 11.5 (SPSS Science, Chicago, IL, USA). The proportion of HUVECs in each phase of the cell cycle was compared between proliferating and quiescent samples using the unpaired *t*-test. The expression of sst was compared between proliferating and quiescent samples using the Mann–Whitney *U*-test. *P*-values <0.05 were considered statistically significant. Data from the WST-1 proliferation assay were analysed using ANOVA with *post hoc* Dunnett's pairwise multiple comparison *t*-tests. *P*-values <0.05 were considered statistically significant.

## RESULTS

### Cell cycle analysis of proliferating and quiescent HUVECs

**Figure 1:**
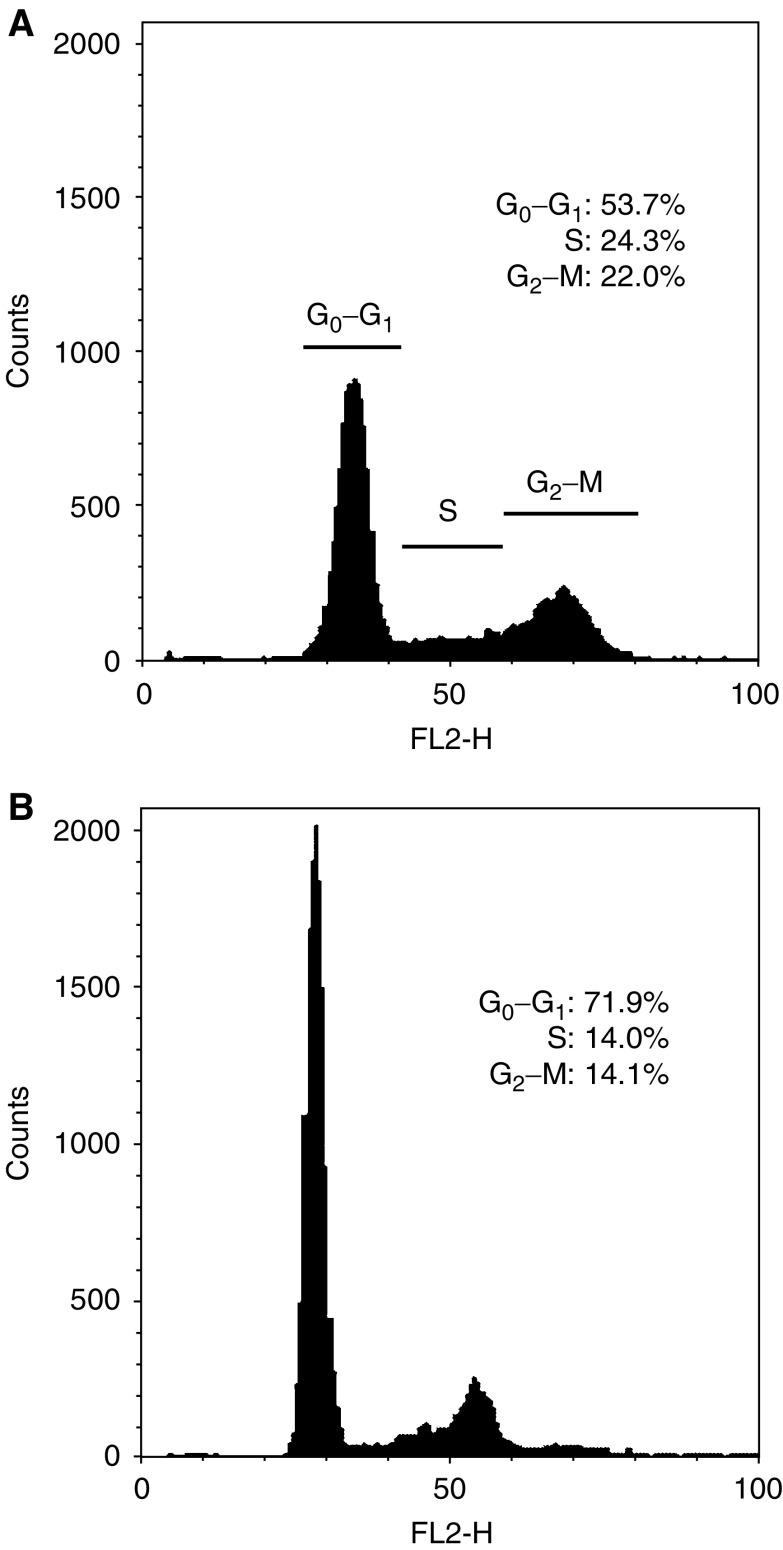
Flow cytometry for cell cycle parameters of propidium–iodide-stained samples of (**A**) proliferative, and (**B**) quiescent HUVECs. This was performed in triplicate for cells derived from each patient.

Quiescent cells demonstrated reduced cell cycle progression in comparison to the corresponding proliferating cells (Figure 1). The proportion of quiescent cells in the S phase of the cell cycle distribution was significantly lower than that of the corresponding proliferating cells (reduced by 18.1±5.8–43.8±2.4%; *P*<0.05; Figure 1). Additionally, significantly more quiescent endothelial cells were distributed in the G_0_–G_1_ phases (increased by 31.9±2.5–73.1±3.2%; *P*<0.05; Figure 1). This indicated that HUVECs subjected to 24 h growth factor deprivation were prevented from progressing from G_0_–G_1_ to S phase. This validated our protocol for the establishment of proliferating and quiescent endothelial cell cultures.

### qRT–PCR of proliferating and quiescent HUVECs

Sst 2 and 5 were preferentially expressed in proliferating cultures (Table 2). There appeared to be no relationship between cell cycle progression and sst 1 or 3 expression (Table 2); therefore, these sst were not quantified. Only one of the quiescent cultures was positive for sst 2, and the expression of sst 2 in this sample was significantly lower than that of the corresponding proliferating culture (*P*<0.01). The quiescent culture from this sample was also positive for sst 5, and again, the expression was significantly reduced (*P*<0.01). Considering that all other quiescent samples were negative for sst 2 and 5, it is possible that this sample contained proliferating cells. Most of the proliferating samples coexpressed at least two receptors, and sst 2 was always coexpressed with at least one other receptor. Sst 1 and 5 were only expressed simultaneously if sst 2 was also expressed. All samples were negative for sst 4.

### Immunohistochemistry

**Figure 2:**
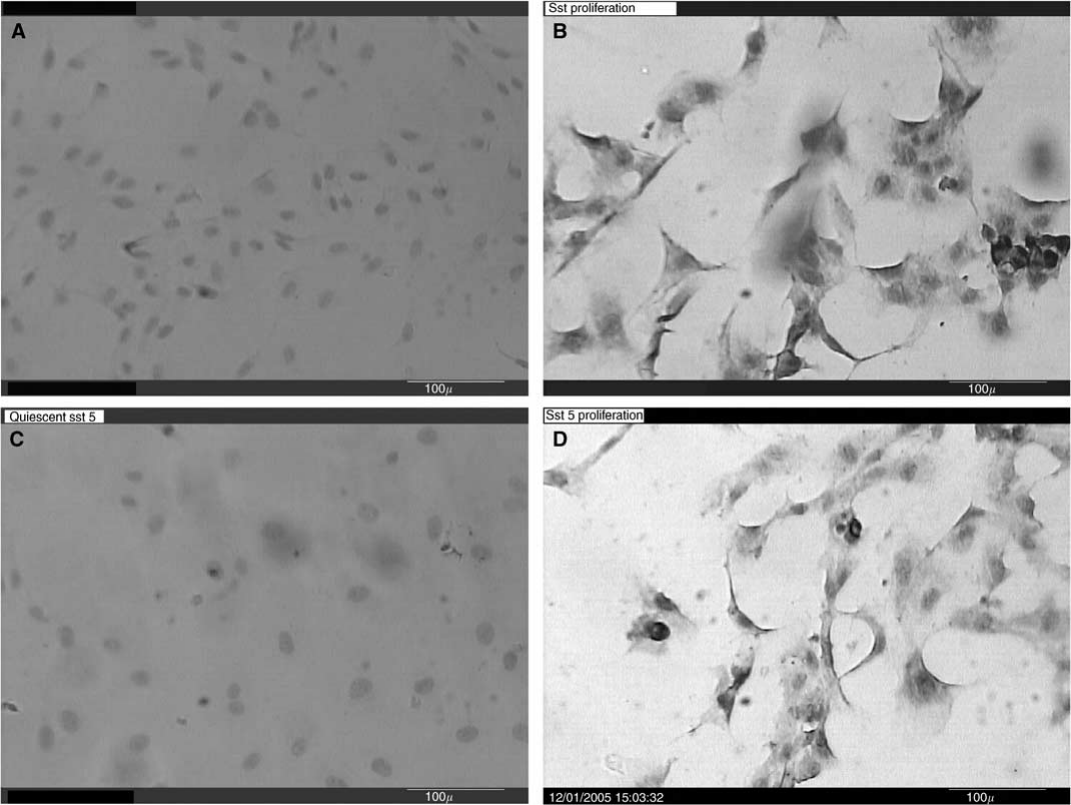
Immunohistochemistry: immunostaining was performed in cultures on chamber slides in quadruplicate. (**A**) Quiescent cells are negative for sst 2 positivity; (**B**) uniform immunopositivity for sst 2 in the proliferating cells; (**C**) quiescent cells are negative for sst 5 positivity; and (**D**) uniform immunopositivity for sst 5 in the proliferating cells (magnification × 10).

Sst 2 and 5 receptor positivity was seen in all proliferating but not quiescent HUVEC culture (Figure 2A and C, quiescent cells are negative for sst 2 and sst 5, respectively; Figure 2B and D, uniform immunopositivity for sst 2 and 5 in the proliferating cells).

### Effect of Octreotide and SOM230 on HUVEC proliferation

**Figure 3:**
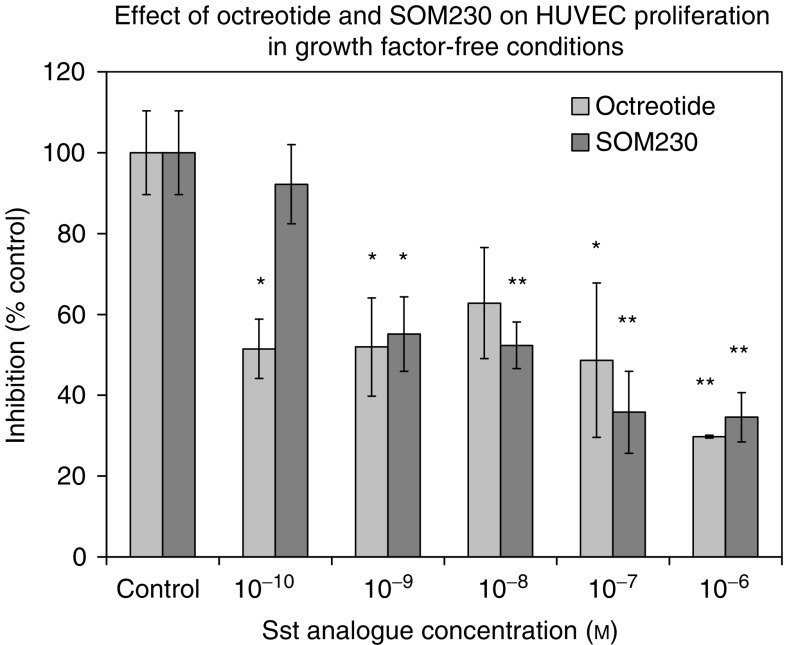
Effect of Octreotide and SOM230 on HUVEC proliferation in growth factor-free conditions. Octreotide significantly inhibited HUVEC proliferation across the concentration range 10^−10^–10^−6^ M (48.5±7.3–70.2±0.4% inhibition), while SOM230 significantly inhibited HUVEC proliferation across the concentration range 10^−9^–10^−6^ M (44.9±9.2–65.4±6.1% inhibition) in a dose-dependent manner. Effects on proliferation were determined using the WST-1 proliferation assay. Data are expressed as mean±s.e.m., determined from six replicates. ^**^P<0.01, ^*^P<0.05.

Octreotide significantly inhibited HUVEC proliferation across the concentration range 10^−10^–10^−6^ M (48.5±7.3–70.2±0.4% inhibition), while SOM230 significantly inhibited HUVEC proliferation across the concentration range 10^−9^–10^6^ M (44.9±9.2–65.4±6.1% inhibition) in a dose-dependent manner (Figure 3).

## DISCUSSION

We have identified that sst 2 and 5 are preferentially expressed in the proliferating phenotype in HUVECs, and that a proportion of HUVECs express sst 1 and 3, irrespective of proliferative status. Our results support previous *in vivo* and *in vitro* findings on the preferential expression of sst 2 in activated endothelial cells (Reubi *et al*, 1994, 1996, 2001; ten Bokum *et al*, 1999; Koizumi *et al*, 2002). The finding that both sst 5 mRNA and positive immunostaining for sst 5 were expressed in HUVECs, and altered with proliferative status, is novel and indicates that both the mRNA and the protein are expressed in proliferation. Sst 5 has not been specifically reported in vessels surrounding tissues with characteristic neovascularisation. In tumours, however, the presence of mRNA for sst 2, sst 5, or for both, has positively correlated with ^125^I-[Tyr^3^]-Octreotide binding sites. In 1998, Siehler *et al* suggested that ^125^I-[Tyr^3^]-Octreotide binding is frequently attributable to sst 5. Considering that ^125^I-[Tyr^3^]-Octreotide has high affinity for sst 2 and moderate affinity for sst 5, it is probable that Octreotide binding previously reported in peritumoral vessels may have been partially due to the presence of sst 5, in accordance with our findings in HUVECs. Sst 5 is preferentially expressed in mitogen-stimulated human T-lymphocytes (Ghamrawy *et al*, 1999), and we have now shown that this preferential expression extends to endothelial cells. Vapreotide (an analogue with sst 2 and 5 activity) has been shown to inhibit proliferation of CCK-stimulated CHO cells, which expressed endogenous CCK receptors and that were transfected with sst 5. The effects of sst 5 appeared to be due to the inhibition of guanylate cyclase, and a consequent reduction in cyclic GMP formation, which modulated the activation of the MAPK cascade (Cordelier P *et al*, 1997). As MAP kinase activation is associated with proliferation of endothelial cells (Bogatcheva *et al*, 2003; Pintus G *et al*, 2003), its inhibition may potentially be the mechanism by which sst 5 activation may have an antiproliferative action.

The finding that HUVEC proliferation was inhibited by both Octreotide and SOM230 indicates the likelihood that sst 2 and 5 are functional in the proliferation of these endothelial cells. However, as both Octreotide and SOM230 have both sst 2 and 5 activities, it is unclear whether it is activation of either or both that is causing the inhibition of proliferation. The recent development of a new generation of somatostatin analogues that target different receptor combinations (Lamberts *et al*, 2002) will aid in further characterisation of the role of sst in the endothelium.

Despite much evidence of the antiproliferative effects of sst 2 activation, the clinical use of Octreotide as an antineoplastic agent has been disappointing (Hejna *et al*, 2002). This may in part be due to the presence of sst 5 on peritumoral vessels. Recently, Zatelli *et al* (2001) showed that sst 5 agonists can inhibit the antiproliferative activity of sst 2 agonists in the human medullary thyroid carcinoma cell line TT. Sst 2 and sst 5 exert antiproliferative effects in the pituitary cell line AtT-20 via similar mechanisms (Tallent *et al*, 1996), yet in CHO cells, the two receptors exert their antiproliferative effects via different mechanisms (Buscail *et al*, 1995). This suggests that the effects of coactivation of sst 2 and 5 are highly tissue specific, and perhaps the antiproliferative effects of sst 2 are antagonised by sst 5 in the endothelium.

We have also shown that HUVECs express sst 1 irrespective of proliferative status. The expression of sst 1 in both proliferating and quiescent endothelium, however, does not eliminate sst 1 as a suitable therapeutic target. In CHO-K1 cells, sst 1 induces cytostatic effects by modulating the MAP kinase pathway (Florio *et al*, 2000). Also, Buchan *et al* (2002) have shown that activation of sst 1 inhibits endothelial cell migration. There has been no evidence to date, however, that sst 1 induces apoptosis. The activation of endothelial sst 1 may therefore inhibit cell migration and induce cytostatic effects in proliferating endothelial cells, without inducing apoptosis in quiescent cells. The expression of sst 1 in quiescent cells also suggests that sst 1 may have other roles in endothelial functions that are not associated with cell cycle progression.

Our results also show that HUVECs express sst 3, in accordance with the findings of Jia *et al* (2003). Only two of our samples, however, expressed this receptor subtype. Florio *et al* (2003) found that sst inhibits DNA synthesis in the sst 3-expressing endothelial cell line Eahy926, and that this effect was blocked by a sst 3 subtype-specific antagonist.

We observed high variability in the coexpression of sst by proliferating HUVECs.

Coexpression of sst 2 and 5 occurred in five of nine proliferating samples. Also, two of nine proliferating samples expressed sst 2 when sst 5 was absent, and one of nine expressed sst 5 when sst 2 was absent. It is possible that there is variation in the temporal expression of sst in HUVECs derived from different sources, and that coexpression of sst 2 and 5 may transiently occur in more samples than is indicated in this study. The cause of this variability is unclear; a similar phenomenon occurs in the immunocytohistochemical detection of sst in tumour vessels from different patients (Reubi *et al*, 1994). Inconsistencies in sst expression are not unique to the endothelium, and variation is evident in a wide range of normal and neoplastic tissues (Hofland and Lamberts, 2001; Reubi *et al*, 2001). This high variability in the coexpression of endothelial sst may be of particular importance in the clinical application of sst analogue therapy.

Overall, these data show that sst may have a functional role in angiogenesis with dynamic changes in sst 2 and 5 expression during proliferation and inhibition of proliferation by the analogues that have sst 2 and 5 activity. Further characterisation of the role of endogenous sst and its receptors in modulating endothelial function in other endothelial cell models may define their role further.

## Figures and Tables

**Table 1:** Sst 1, 2, 3, 4, 5 oligonucleotide forward primers, reverse primers and internal probes

SSTR 1	Forward primer	5′-GCTCGGAGCGCAAGATCA-3′
	Reverse primer	5′-CGTCGTCCTGCTCAGCAAA-3′
	Probe	5′-CTTAATGGTGATGATGGTGGTGATGGTGTTT-3′
		
SSTR 2	Forward primer	5′-TGGTCCACTGGCCCTTTG –3′
	Reverse primer	5′-TTGATGCCATCCACAGTCATG-3′
	Probe	5′-CAAGGCCATTTGCCGGGTGG-3′
		
SSTR 3	Forward primer	5′-TGGGCCTGCTGGACTC-3′
	Reverse primer	5′-GTTGAGGATGTAGACGTTGGTGACT-3′
	Probe	5′-CCGTGTGCCGCAGGACCACA-3′
		
SSTR 4	Forward primer	5′-GCGCTCGGAGAAGAAAATCA-3′
	Reverse primer	5′-GGCTGGTCACGACGAGGTT-3′
	Probe	5′-CGTCTTTGTGCTCTGCTGGATGCCTT-3′
		
SSTR 5	Forward primer	5′-TCATCCTCTCCTACGCCAACA-3′
	Reverse primer	5′-TGGAAGCTCTGGCGGAAGT-3′
	Probe	5′-CCGTCCTCTCAGGCTTCCTCTCGGA-3′

Sst=somatostatin; SSTR=somatostatin receptor.

**Table 2:** Expression of sst 1, 2 and 5 in proliferating and quiescent HUVECs, including relative expression of sst 2 and 5

	**Receptor**							
	**SSTR 1**		**SSTR 2**		**SSTR 3**		**SSTR 5**	
**Patient**	**P**	**Q**	**P**	**Q**	**P**	**Q**	**P**	**Q**
1	−	+	+	−	−	+	+	−
2	+	+	+	−	−	−	+	−
3	+	+	+	−	−	−	−	−
4	+	+	+	−	−	−	−	−
5	+	+	+	−	−	−	+	−
6	+	−	−	−	−	−	−	−
7	−	−	+	−	+	−	+	−
8	−	+	−	−	−	−	+	−
9	−	−	100±43.0%	12.2±8.5%	−	−	100±10.7%	1.1±1.4%

P=proliferative; Q=quiescent; HUVEC=human umbilical vein endothelial cell; SSTR=somatostatin receptor.

Experiments were performed for six replicates on each of the cultures derived from the nine patients.

Relative sst gene expression is displayed as a percentage of the corresponding proliferative cells. Each quantified value is expressed as mean±s.d.
